# Editorial: Innovation in minimally invasive therapies, biosensing, and screening: Data-driven models, complex networks, and experiments

**DOI:** 10.3389/fmedt.2023.1146068

**Published:** 2023-02-07

**Authors:** Sundeep Singh, Roderick Melnik, Ramjee Repaka, Paola Saccomandi

**Affiliations:** ^1^ Faculty of Sustainable Design Engineering, University of Prince Edward Island, Charlottetown, PEI, Canada; ^2^ MS2Discovery Interdisciplinary Research Institute, Wilfrid Laurier University, Waterloo, ON, Canada; ^3^ BCAM - Basque Center for Applied Mathematics, Bilbao, Spain; ^4^ Department of Mechanical Engineering, Indian Institute of Technology Ropar, Punjab, India; ^5^ Department of Mechanical Engineering, Politecnico di Milano, Milan, Italy

**Keywords:** minimally invasive therapies, biosensors and smart sensor systems, mathematical modelling and simulations in diagnostic and therapeutic, multiscale, bio-networks and data-driven models, artificial intelligence and machine-learning algorithms

**Editorial on the Research Topic** Innovation in minimally invasive therapies, biosensing, and screening: Data-driven models, complex networks, and experiments

This Research Topic presents a collection of novel modelling, computational, experimental, and clinical studies in the field of minimally invasive diagnostics, and disease treatments targeting cancer, cardiovascular diseases, neurodegenerative disorders and respiratory diseases.

In the first article of this Research Topic, Johnson et al. presented an anatomically and physiologically accurate cardiovascular flow simulator that can be used as an educational tool for training medical professionals as well as testing medical devices for cardiac catheterization. The developed cost-effective simulator was operated by Arduino programming and includes an anatomical right atrium/ventricle, femoral and radial access sites, and considers the variability of arm position and human hemodynamics. The authors have successfully demonstrated the catheter insertion simulation utilizing the developed cardiovascular flow simulator, highlighting the significant potential of attaining low-cost catheter design iteration before moving to animal trials.

Leveraging fundamental research in physiology, Zaid et al. presented a mechanism-driven modelling framework to develop effective and non-invasive methods for monitoring cardiac function *via* ballistocardiography. Notably, connections were established between changes in ballistocardiography signals, pressure-volume loops and cardiac function. Theoretical predictions based on a mechanism-driven cardiovascular model have been compared with experimental studies conducted on swine. The presented results clearly provide experimental evidence that mechanism-driven modelling can be utilized as a guide to interpreting cardiovascular signals that can be further extended to study heart failure, valvular stenosis, sepsis, and atherosclerosis.

In another study of this Research Topic, Iqbal et al. explored the feasibility of unsupervised machine learning classification methods for physiological stress detection. Different unsupervised learning clustering classifiers such as affinity propagation, balanced iterative reducing and clustering using hierarchies (BIRCH), K-mean, mini-batch K-mean, mean shift, density-based spatial clustering of applications with noise (DBSCAN) and ordering points to identify the clustering structure (OPTICS) have been comparatively analysed. The reported results demonstrate the potential of these unsupervised machine learning classifiers for the development of non-invasive detection and monitoring of physiological and pathological stress through wearable devices.

Staelens et al. reported the results of *in vitro* experimental investigations involving low-intensity near-infrared photobiomodulation of living cells, tubulin and microtubules. The authors presented the results from a series of experiments to investigate the cellular and sub-cellular effects of photobiomodulation that could lead to designing more precisely effective photobiomodulation parameters for treating neurodegenerative disorders.

Correvon et al. reported a benchmark study to evaluate the impact of the additional high-efficiency particulate air filter on the automatic positive airway pressure (APAP) performance and continuous positive airway pressure level in simulated sleep apnea events. Four different antibacterial filters on APAP mode performance with and without added unintentional air leaks were tested for two simulated respiratory events (i.e., obstructive apnea and hypopnea events).

The paper by Tuszynski and Costa reviews the existing and developing applications of low-energy amplitude-modulated radiofrequency electromagnetic fields for the systemic treatment of cancer. Authors also propose a mechanism of action of low-energy amplitude-modulated radiofrequency electromagnetic fields at cellular and subcellular levels (involving microtubules, ionic flows and changes in cellular metabolism) focussed on the treatment of advanced hepatocellular carcinoma.

This Research Topic brings together five research articles and one review paper that highlights novel findings, the current state-of-the-art developments, and provides an outlook for further investigations in the interdisciplinary research field of application and integration of computational models in biomedical engineering, based on multiscale, multiphysics, and bio-networks data-driven approaches. We believe that the presented knowledge in this Research Topic will provide a future pathway for ground-breaking research in the field of minimally invasive therapies, biosensing, and screening.

